# University College Hospital

**Published:** 1906-11-10

**Authors:** 


### UNIVERSITY COLLEGE HOSPITAL.

### THE COMPLETION OF THE BUILDINGS.

A large company at University College Hospital welcomed the Duke and Duchess of Connaught last Tuesday on the occasion of their opening the new building, erected through the generosity of the late Sir John Blundell Maple. They included the Duke of Bedford (President of the Hospital), the Duchess of Devonshire, Lord Reay (a Vice-President of the Hospital and President of University College), Lady Reay, Lord Monkswell (Treasurer), Lady Monkswell, the Bishop of London, Lady Ormonde, Mr. Henry Lucas (Chairman of the Hospital Committee and of the Rebuilding Committee), Sir T. Barlow (Senior Physician), Lady Barlow, Sir A. Rücker, Dr. Gregory Foster, Sir A. Prevost, Sir J. Reid, Mr. W. Baily, Mr. A. E. Barker (Senior Surgeon), Mrs. Montague Ballard (wife of the late Sir J. Blundell Maple and a Vice-President of the Hospital), Baroness Hermione von Eckardstein, Mr. Paul Waterhouse (architect), Sir William and Lady Gowers, Sir Victor and Lady Horsley, Sir J. Rotton, Colonel Sir Collin Scott-Moncrieff, S. B. L. Cohen, the Hon. Lady Reid, Sir Benjamin and Lady Franklin, and Mr. Newton H. Nixon (Secretary).

The Duke of Bedford, as President of the Hospital, presented an Address from the Hospital Committee and Medical Staff. The address pointed out that the new building provides for the reception of nearly 300 patients, chiefly in large wards containing 24 beds. The special features of the wards, which were constructed according to the ideas of the late Dr. George Vivian Poore, who unfortunately only saw the commencement of a work the necessity of which he had long urged, are of great benefit, not only in the treatment of the sick, but also in preserving the health of the nurses. The isolation of the wards, cut off, as they are, from the central block and from the sanitary portion of the wing, the cross-ventilation by large windows, their warmth in winter and coolness in summer, are all improvements on the old hospital wards, and lead to greater success in the treatment of disease. It was considered of advantage to remove the patients suffering from enteric fever from the general wards, and to place them in wards devoted entirely to the treatment of that disease. This arrangement has proved a success both in diminishing the risk of infection, which undoubtedly occurred when the patients were treated in the general wards, and in making efficient the methods of disinfection which are necessary in such cases. The advances made in the application of various forms of electricity in disease has necessitated the establisment of electro-therapeutic and *x*-ray departments.

Lord Reay next addressed the Duke of Connaught on behalf of the Council of University College, and mentioned the names of some of the distinguished practitioners who had shed a lustre on the Hospital in the past—Parkes, Cooper, Sir Robert Liston, who performed the first operation in England under chloroform anæsthesia on December 21, 1846, Erichsen, Thomas Wharton Jones, and others, and expressed the pleasure of the Council that by their presence their Royal Highnesses had given such valuable encouragement to those who labour in this field with so much devotion.

The Duke of Connaught said :—" Your Graces, my Lords, Ladies and Gentlemen,—I beg to thank his Grace the Duke of Bedford and Lord Reay very much for the addresses which they have just read to me. This hospital and University College are so closely associated, as shown by the two addresses delivered by the President of the Hospital and the President of University College, that I [illegible] ask to be forgiven if, in answering them both at once, I mix up a little the hospital and the college. It is a satisfaction to me to have been asked here on this occasion, and to enable the Duchess, as well as myself, to associate ourselves with this opening of the new University College Hospital. We have heard from the remarks that fell from the Duke of Bedford how this hospital took its beginning, and how, with an increased number of in-patients, with increased work, and with increased advance in medical science, a necessity arose for a new hospital more suited to the modern day. This new hospital we are now standing in at this moment. I am happy to think that his Majesty the King, as Prince of Wales, was associated with this hospital in having laid the foundation-stone. I notice that her gracious Majesty our late beloved Queen was patron of this hospital from the year 1837 till the time of her death, and I congratulate the hospital that our present Sovereign is still at its head. These two facts, which I just mention, and which you have so kindly applauded, show for themselves the great value and the great store that is laid on the importance of this hospital, as well as of University College. I notice among the facts connected with it that 65,000 in- and out-patients have annually been treated in this hospital. That is a vast number, and one wonders how they could have been treated without a new hospital more suited to the enormous demands made at the present day. I think, and we all think, that this hospital owes a deep debt of gratitude to the late Sir Blundell Maple. His noble generosity in giving £200,000 towards this hospital has enabled those who are deeply interested to carry out this most important and, from all accounts, most carefully thought-out and most admirable building. May I congratulate the architect, and the father of the architect, who originally drew out the designs for this hospital, on the great success with which this work has been carried out, of which this noble hall in which we are standing is one of the most beautiful buildings. Ladies and gentlemen, hospitals in London are many, they are large, they are magnificent, and they are well-supported, but the demands for hospitals in this metropolis are even greater than we are able to meet. Therefore we must feel very grateful to those with whom rests the administration of a hospital like this that they have been enabled to construct one which is so suitable to what is required in the present day, and which I am certain will have a great future before it in the amount of good that it will do and the amount of suffering that it will mitigate. Professors and collegiates of University College Hospital, let me tell you what a pleasure it is to me to meet you. You have heard from Lord Reay the distinguished services rendered by many who have gone before you, whose names will ever live in English medical science, and probably in the science of this world. You, therefore, follow men who have thrown a great lustre on the medical profession, men who have been a blessing to this world, and who have done immense things to mitigate the sufferings which are ever among us. Let me hope that those whom I have the honour of addressing will rise to follow in the footsteps of the great men who have preceded them, and that they will ever remember that the public generously recognises the skill, the devotion, and the sympathy shown by our medical men. We in England hope that we are even more sympathetic with others, and especially those in suffering, than other countries, and I am certain that the rising members of the medical profession will ever do all they can to keep up the noble attributes of the English doctor in nobly devoting themselves to the grand profession, the science of which is increasing in importance and in interest every year. I thank you, your Grace and Lord Reay, for the very interesting and very kind addresses you have just read to me.

The Bishop of London then offered prayers, after which the Duke of Connaught declared the hospital open.

